# Sex differences in pre- and post-synaptic glutamate signaling in the nucleus accumbens core

**DOI:** 10.1186/s13293-023-00537-4

**Published:** 2023-08-18

**Authors:** Melissa C. Knouse, Andre U. Deutschmann, Miroslav N. Nenov, Mathieu E. Wimmer, Lisa A. Briand

**Affiliations:** 1 Department of Psychology, Temple University, Weiss Hall, 1701 North 13th Street, Philadelphia, PA 19122 USA; 2 Neuroscience Program, Temple University, Weiss Hall, 1701 North 13th Street, Philadelphia, PA 19122 USA

**Keywords:** Nucleus accumbens, Sex differences, Glutamate, PKMζ, Synaptic plasticity, Long-term depression

## Abstract

**Background:**

Glutamate signaling within the nucleus accumbens underlies motivated behavior and is involved in psychiatric disease. Although behavioral sex differences in these processes are well-established, the neural mechanisms driving these differences are largely unexplored. In these studies, we examine potential sex differences in synaptic plasticity and excitatory transmission within the nucleus accumbens core. Further understanding of baseline sex differences in reward circuitry will shed light on potential mechanisms driving behavioral differences in motivated behavior and psychiatric disease.

**Methods:**

Behaviorally naïve adult male and female Long-Evans rats, C57Bl/6J mice, and constitutive PKMζ knockout mice were killed and tissue containing the nucleus accumbens core was collected for ex vivo slice electrophysiology experiments. Electrophysiology recordings examined baseline sex differences in synaptic plasticity and transmission within this region and the potential role of PKMζ in long-term depression.

**Results:**

Within the nucleus accumbens core, both female mice and rats exhibit higher AMPA/NMDA ratios compared to male animals. Further, female mice have a larger readily releasable pool of glutamate and lower release probability compared to male mice. No significant sex differences were detected in spontaneous excitatory postsynaptic current amplitude or frequency. Finally, the threshold for induction of long-term depression was lower for male animals than females, an effect that appears to be mediated, in part, by PKMζ.

**Conclusions:**

We conclude that there are baseline sex differences in synaptic plasticity and excitatory transmission in the nucleus accumbens core. Our data suggest there are sex differences at multiple levels in this region that should be considered in the development of pharmacotherapies to treat psychiatric illnesses such as depression and substance use disorder.

## Plain Language Summary

Understanding normal neural signaling within the nucleus accumbens, a key brain region involved in psychiatric disease including substance use disorder and depression, could provide insight into treatment options for these disorders. Although we know the behaviors regulated by the nucleus accumbens can differ between males and females, we do not understand the underlying differences in brain processing that could contribute to these behavioral differences. Further, even in cases when these behaviors are not different, the underlying brain signaling may exhibit sex-specific mechanisms. The current studies examined excitatory signaling with the nucleus accumbens in both rats and mice at the level of both individual cells and circuits. We found that female rodents (rats and mice) exhibit higher levels of excitatory signaling within the nucleus accumbens than male rodents. Further, procedures that can dampen neural transmission in males are not sufficient to do so in females, suggesting that excitatory signaling in the nucleus accumbens of females is less plastic. Finally, our last set of studies utilized mice missing the protein, PKMζ, and demonstrated that this reversed some of the sex differences seen in normal mice, pointing to a critical role for this protein in maintaining these differences. Our data suggest there are sex differences at multiple levels in this region that should be considered in the development of pharmacotherapies to treat psychiatric illnesses such as depression and substance use disorder.

## Highlights

- Female Long-Evans rats and C57Bl/6J mice have a heightened AMPA/NMDA ratio and larger readily releasable pool of glutamate in the nucleus accumbens core compared to males.
- Long-term depression in the nucleus accumbens core induced by 5 min of low-frequency stimulation (LFS) functions in a sex-specific manner and is modulated by PKMζ.
- There are sex differences in multiple measures of excitatory transmission within the nucleus accumbens core that may play a role in psychiatric disease.

## Introduction

Dysregulated glutamate signaling in the brain is involved in psychiatric disease [1–4]. Many of these diseases, such as depression and substance use disorder (SUD), present differently in males and females [5–13]. Despite these well-established sex differences, the extent to which baseline glutamate transmission differs between the sexes is not fully understood. There are sex differences in glutamate levels and receptor expression in regions including the hippocampus and prefrontal cortex [14–16]. Further, we previously demonstrated females exhibit heightened glutamatergic transmission in the medial prefrontal cortex (mPFC) compared to males [17]. Together, these data highlight the possibility there are baseline sex differences in excitatory transmission that may underlie behavioral sex differences in psychiatric disease.

The nucleus accumbens (NAc), a brain region within the striatum, plays a critical role in motivated behavior and is involved in diseases with dysregulated motivation and reward processing [18–26]. The NAc is part of the mesocorticolimbic reward system, receiving glutamatergic input from the prefrontal cortex, amygdala, medial thalamus, and hippocampus. The NAc has low levels of estrogen receptors (ERs) and they are localized to extranuclear sites [27, 28]. Despite this, estrogens are proposed to work through glutamatergic mechanisms in this region to modify neurophysiology and behavior [28–33]. Estradiol decreases AMPA receptor (AMPAR) binding in the NAc and decreases spine density specifically within the core subregion [29, 34, 35]. Miniature excitatory postsynaptic current (mEPSC) frequency and amplitude vary across the estrous cycle and bath application of estradiol decreases mEPSC frequency in the NAc core of females but not males [36–38]. Behaviorally, metabotropic glutamate receptor 5 is necessary for estradiol-induced alterations to anxiety and cocaine-taking [33, 39]. Together, these studies demonstrate ovarian hormones can modulate plasticity within the reward system. Further, they highlight the likelihood that biological sex could alter overall excitatory transmission within the NAc.

Indeed, there are established sex differences in plasticity within this region [40]. Distal dendritic spine density and the proportion of large spines on medium spiny neurons (MSNs) in the NAc are greater in females than males [41, 42]. Overall spine synapse density is not significantly different between the sexes in the NAc core, but females have higher spine synapse density in caudal blocks of the core than males [43]. Further, mEPSC frequency is higher in both prepubertal and adult female MSNs in the NAc core than in males [42, 44]. Previous work from our lab found females have a significantly larger readily releasable pool (RRP) of glutamate and lower release probability within the NAc core compared to males [45]. Overall, these data indicate that sex differences in glutamate signaling exist within the NAc and females may have heightened glutamatergic transmission in this region compared to males.

Trafficking of AMPARs specifically underlies many of the psychiatric diseases involving dysregulated glutamate signaling [1, 46–51]. PKMζ, a constitutively active isoform of Protein Kinase C, is an AMPAR trafficking protein that potentiates NSF-mediated insertion of GluA2-containing AMPARs to the cell membrane [52]. This makes PKMζ an interesting target for studies on the synaptic plasticity underlying learning, memory, and motivated behavior. PKMζ is upregulated following performance of learning and memory tasks and cocaine experience [53–55]. Further, PKMζ is involved in depression- and anxiety-like behavior and drug-taking [53, 56–59].

Interestingly, biological sex influences the role of PKMζ in these behaviors. While constitutive PKMζ knockout potentiates cocaine-taking in both sexes, site-specific knockout in the NAc potentiates cocaine-taking exclusively in males [53]. Constitutive PKMζ knockout also reduces anxiety-like behaviors in male, but not female, mice [58]. These studies indicate PKMζ influences these behaviors in a sex-specific manner. Therefore, further characterization of the role of PKMζ in synaptic plasticity within the reward system will aid in our understanding of the mechanisms that may drive sex differences in psychiatric disease.

The aim of these experiments was to better characterize baseline sex differences in glutamate signaling in the reward system. As we previously found significant sex differences in the size of the RRP of glutamate in the NAc core, we chose to further explore a variety of electrophysiological measures within this region. Here, we found significant sex differences in both pre- and post-synaptic excitatory transmission. In combination with our previous findings in the mPFC [17], these data suggest there are functional sex differences at many levels within the mesocorticolimbic reward system. Gaining a better understanding of these differences could provide insight into sex-specific treatments for disorders involving dysregulated motivation and reward behavior.

## Methods

### Subjects

Wildtype studies: Animals were between 2 and 5 months old at time of use. Male and female Long-Evans rats and C57Bl/6J mice were bred in house for electrophysiology experiments. Constitutive PKMζ deletion: the current study used PKMζ knockout mice as described previously [60]. Heterozygous PKMζ KO mice on a C57BL/6J background were mated resulting in mutant and wildtype littermates. Animals were group housed throughout the experiments with food and water available ad libitum. All animals were housed in a temperature- and humidity-controlled animal care facility. Mice had a 12 h light/dark cycle (lights on at 7:00 A.M.) and rats had a 12 h light/dark cycle (lights off at 8:30 A.M.). Estrous cycle was not monitored throughout these studies. All procedures were approved by the Temple University Animal Care and Use Committee.

### Slice preparation

Mice were decapitated following cervical dislocation and rats were decapitated following isoflurane anesthesia. The brain was removed and coronal slices (250 μm for mice, 300 μm for rats) containing the nucleus accumbens were cut with a Vibratome (VT1000S, Leica Microsystems) in an ice-cold artificial cerebrospinal fluid solution (ACSF), in which NaCl was replaced by an equiosmolar concentration of sucrose. ACSF consisted of (in mM): 128.2 NaCl, 2.9 KCl, 1.2 MgSO_4_7H_2_O, 1.25 NaH_2_PO_4_, 28.8 NaHCO_3_, 2 CaCl_2_, 10 glucose (pH 7.2–7.4 when saturated with 95% O_2_/5% CO_2_). Slices were incubated in ACSF at 32–34 °C for 25 min and kept at 22–25 °C thereafter, until transferred to the recording chamber. The osmolarity of all solutions was 300–315 mOsm. Slices were viewed using infrared differential interference contrast optics under an upright microscope (Slice Scope Pro, Scientifica) with a 40× water-immersion objective.

### Electrophysiology

The recording chamber was continuously perfused (1–2 ml/min) with oxygenated ACSF heated to 32 ± 1 °C using an automatic temperature controller (Warner 278 Instruments). Picrotoxin (100 μM) was added to all solutions to block the GABA_A_ receptor-mediated currents. Recording pipettes were pulled from borosilicate glass capillaries (World Precision Instruments) to a resistance of 4–7 MΩ when filled with the intracellular solution (whole-cell recordings) or to a resistance of 1–2 MΩ when filled with extracellular solution (field recordings). All recordings were conducted with a MultiClamp700B amplifier (Molecular Devices). *Whole-cell recordings.* Intracellular solution contained (in mM): 100 CsCH_3_O_3_S, 50 CsCl, 3 KCl, 0.2 BAPTA, 10 HEPES, 1 MgCl_2_, 2.5 phosphocreatine-2Na, 2Mg-ATP, 0.25 GTP-Tris pH 7.2–7.3 with CsOH, osmolarity 280–290 mOsm). For all measures, cells from at least 3 animals, within each group, were used. Recordings were taken from cells within the nucleus accumbens core. *sEPSCs.* Recordings were conducted in whole-cell voltage-clamp mode (Vh = − 70 mV). Currents were low-pass filtered at 2 kHz and digitized at 20 kHz using a Digidata 1440A acquisition board and pClamp10 software (both from Molecular Devices). Access resistance (10–32 MΩ) was monitored throughout the recordings by injection of 10 mV hyperpolarizing pulses and data were discarded if access resistance changed by > 25% over the course of data acquisition. sEPSCs were detected using sliding-template-based algorithm in pClamp 10. This method compares the shape of the detected current to that of a template. The threshold for the minimum event detected by the template was estimated by the noise analysis of the trace. A route means squared analysis of the noise was performed using ClampFit 10 to confirm that there were no differences between the groups in the amount of noise in the recordings [mean ± SEM, male rats: 1.69 ± 0.19; female rats: 1.86 ± 0.17; male mice: 1.79 ± 0.25; female mice: 1.58 ± 0.15]. Event threshold for sEPSC analysis with a template was set as two times the RMS and all detected events were verified by visual confirmation of a fast rise time and slower exponential decay to baseline. Mean sEPSC amplitude was analyzed from an average sEPSCs trace computed from 150 individual sEPSCs. Mean sEPSC frequencies were analyzed from 180-s-long trace segments. Cumulative probability curves were generated using 150 events from each cell. *AMPA/NMDA ratio*. Evoked responses were triggered by 300 μs constant-current pulses generated by an A310 Accupulser (World Precision Instruments) and delivered at 0.1 Hz via a glass capillary electrode filled with ACSF. The stimulation electrode was positioned within 100 μm of the recorded cell. The amplitude of the current pulses was controlled by a stimulus isolator (WPI Linear Stimulus Isolator A395) and was adjusted to elicit monosynaptic responses in the range of 100–300 pA (the required stimulus intensity ranged from 15 to 80 μA). 1 mM QX-314 was added to intracellular solution for these recordings. Current ratios were computed by dividing the mean peak sEPSC at − 70 mV (AMPA-mediated) by the mean amplitude at + 40 mV, 35 ms after the peak over a 2 ms window (NMDA-mediated). *Readily releasable pool*. After obtaining a stable baseline at − 70 mV, a 100-Hz train was applied for 10 s. EPSC amplitudes were measured by subtracting the baseline current just preceding an EPSC from the subsequent peak of the EPSC. For the RRPtrain technique, EPSC amplitudes were then summed throughout the train stimulus to give a cumulative EPSC curve. A straight line was fitted to the final 15 points of the cumulative EPSC and back-extrapolated to the y-axis. The y-intercept corresponds to RRPtrain, and ptrain = EPSC0/RRPtrain. *Field recordings.* A glass capillary electrode filled with ACSF was placed within 100–300 μm from the recording electrode and used to stimulate excitatory afferents at 0.1 Hz. The field recordings were performed within the nucleus accumbens core. *Long-term depression.* The amplitude of current pulses was set at the intensity required to evoke a 70% maximal response. After 10 min of stable responding, LTD was induced using a paired-pulse protocol (50 ms inter-pulse interval) consisting of a 1-Hz train of paired stimuli for 5 or 10 min. Both the field EPSP (fEPSP) slope (calculated over 1 ms after peak) and fEPSP amplitude were measured (graphs depict slope) from fEPSPs recorded at 0.05 Hz for 60 min following the pairing protocol.

### Data analysis

All analyses were performed using GraphPad Prism 9.0 software (GraphPad Software). Data were analyzed using two-tailed Student’s *t*-test or two-way ANOVA with Sidak’s post hoc tests as appropriate. Kolmogorov–Smirnov tests were performed on the cumulative probability curves. Statistical significance for all tests was set at *α* = 0.05.

## Results

### Female rats and mice have a heightened AMPA/NMDA ratio within the nucleus accumbens core compared to males

**Fig. 1:**
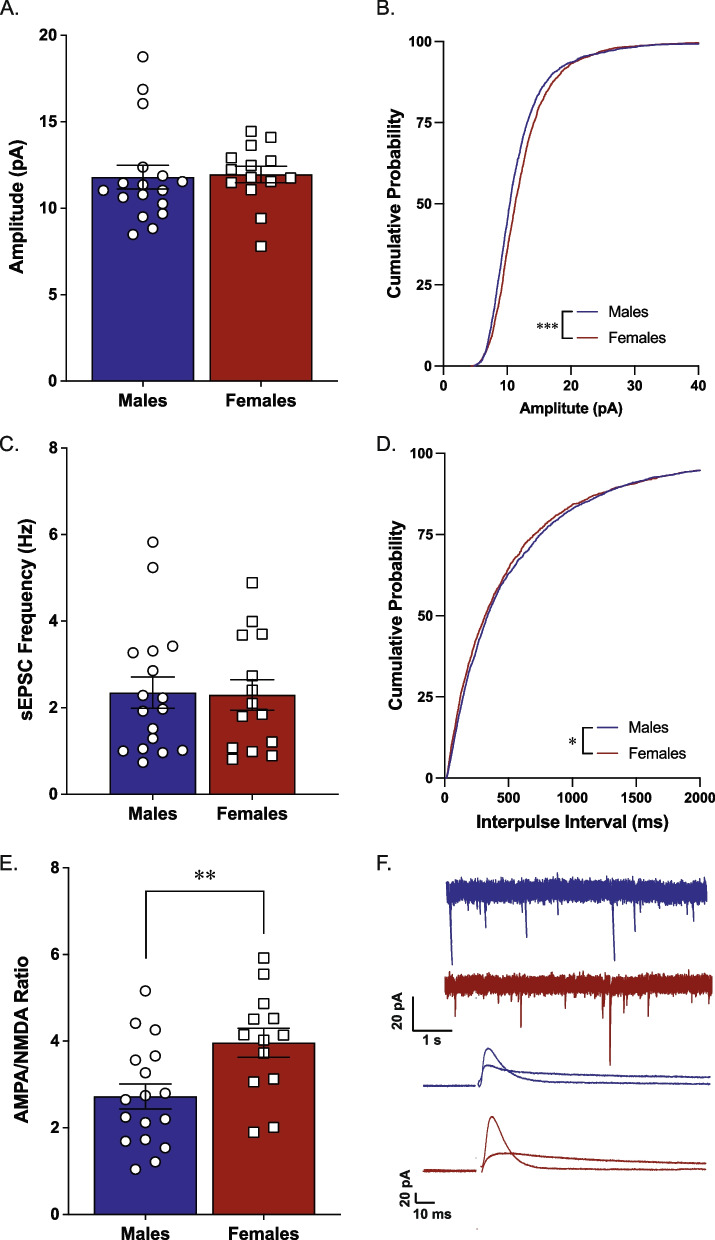
AMPA/NMDA ratio in the nucleus accumbens core is higher in female Long-Evans rats compared to male rats. Whole-cell recordings demonstrated no significant effect of sex on sEPSC amplitude (**A**, **B**; *n* = 11–12/group) or frequency (**C**, **D**; *n* = 10–11/group). Female rats have a significantly higher AMPA/NMDA ratio in this region than male rats (**E**; *n* = 13–17/group). Representative traces for sEPSC recordings and AMPA and NMDA currents (**F**). **p < 0.01 effect of biological sex

**Fig. 2:**
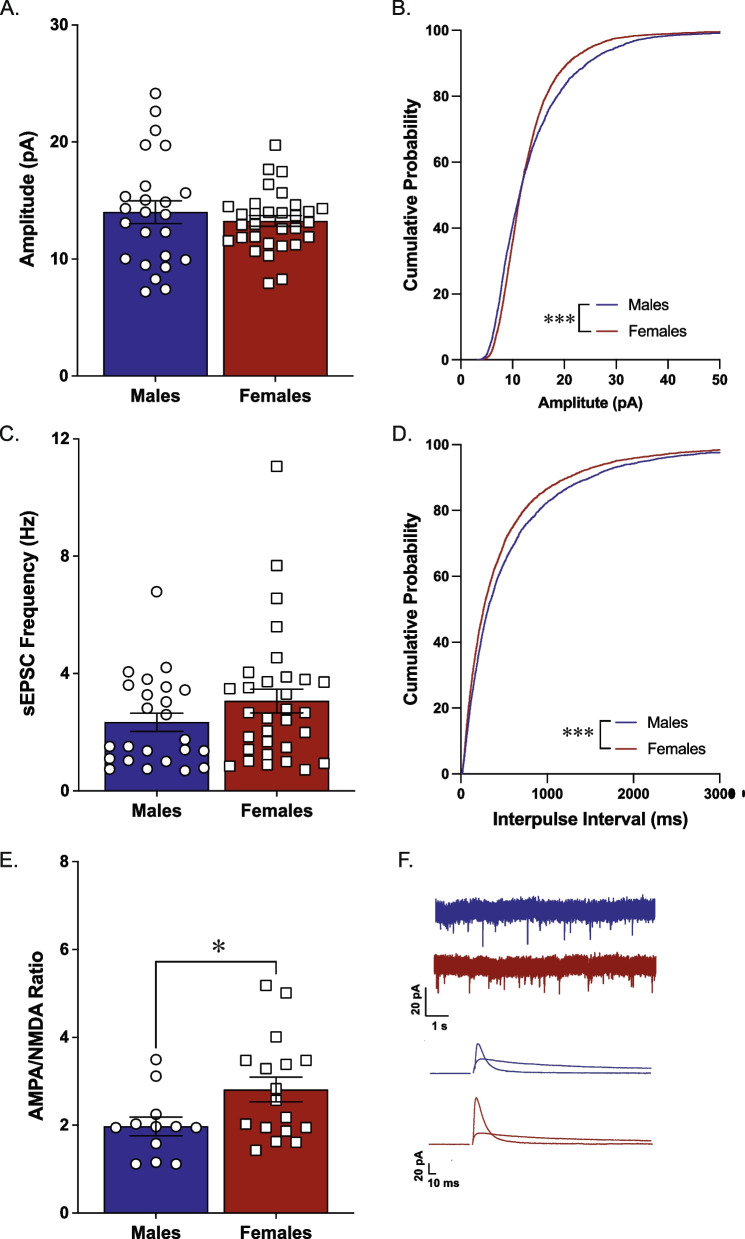
AMPA/NMDA ratio in the nucleus accumbens core is higher in female C57Bl6/J mice compared to male mice. Whole-cell recordings demonstrated no significant effect of sex on sEPSC amplitude (**A**, **B**; *n* = 19–24/group) or frequency (**C**, **D**; *n* = 18–22/group). Female mice have a significantly higher AMPA/NMDA ratio in this region than male mice (**E**; *n* = 12–17/group). Representative traces for sEPSC recordings and AMPA and NMDA currents (**F**). *p < 0.05 effect of biological sex

We examined sEPSC amplitude and frequency and AMPA/NMDA ratio within the NAc core of naïve adult male and female Long-Evans rats. We did not see any significant differences between male and female rats in mean sEPSC amplitude or frequency [*t*(29) = 0.18, *p* = 0.85, *n* = 14–17/group, Fig. 1A; *t*(29) = 0.10, *p* = 0.92, *n* = 14–17/group, Fig. 1C]. Analysis of the cumulative distribution revealed small but significant differences in sEPSC amplitude (K-S *D* = 0.11; *p* < 0.0001; Fig. 1B) and inter-event interval (K-S *D* = 0.043, *p* = 0.027; Fig. 1D). Further, there were no sex differences in sEPSC kinetics, with male rats and female rats exhibiting similar rise (mean ± SEM, males: 2.96 ± 0.14; females: 3.22 ± 0.08) and decay (mean ± SEM, males: 10.0 ± 0.31; females: 10.4 ± 0.18) times. There was, however, a significant difference in AMPA/NMDA ratio between females and males, with females exhibiting a higher ratio [*t*(28) = 2.814, *p* = 0.0088, *n* = 13–17/group; Fig. 1C]. To determine whether these differences in glutamate signaling were present across multiple species, we examined sEPSCs and AMPA/NMDA ratio in naïve male and female C57BL/6J mice. Analysis of the mean sEPSC amplitude [*t*(53) = 0.75, *p* = 0.45; Fig. 2A] and frequency [*t*(53) = 1.35, *p* = 0.18; Fig. 2C] did not reveal any significant sex differences. However, analysis of the cumulative distribution revealed small but significant differences in sEPSC amplitude (K-S *D* = 0.084; *p* < 0.0001; Fig. 2B) and inter-event interval (K-S *D* = 0.065, *p* < 0.0001; Fig. 2D). There were no sex differences in sEPSC kinetics, with male mice and female mice exhibiting similar rise (mean ± SEM, males: 2.39 ± 0.12; females: 2.36 ± 0.08) and decay (mean ± SEM, males: 9.28 ± 0.29; females: 9.55 ± 0.23) times. Similar to what was seen in rats, female mice exhibit higher AMPA/NMDA ratios compared to male mice [*t*(27) = 2.2, *p* = 0.036, *n* = 12–17/group; Fig. 2E].

### Females have a larger readily releasable pool of glutamate in the nucleus accumbens core compared to males

**Fig. 3:**
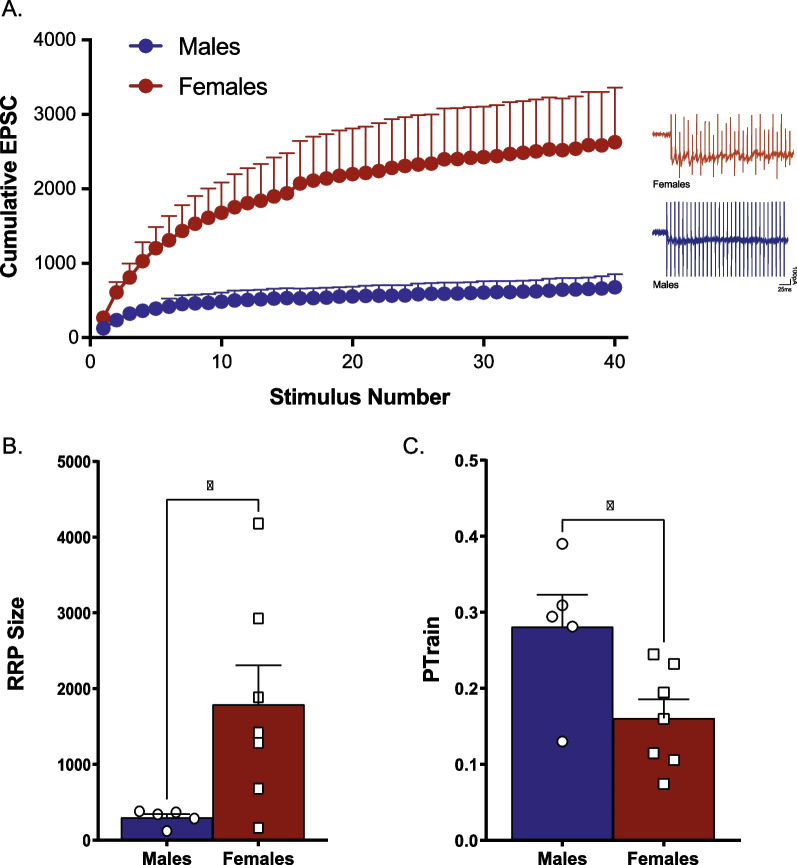
Female mice have a larger readily releasable pool of glutamate and lower release probability in the nucleus accumbens core than male mice. Cumulative EPSC obtained following a 100-Hz train demonstrates a larger response in females compared to males (**A**; *n* = 6–7/group). Analysis of the size of the readily releasable pool (RRP) reveals that female mice have a larger RRP of glutamate compared to male mice (**B**; *n* = 5–7/group). Female mice also exhibit a lower release probability than male mice (**C**; *n* = 5–7/group). *p < 0.05 effect of biological sex

To determine whether the postsynaptic alterations in glutamate transmission were accompanied by differences in presynaptic glutamate transmission, we examined the size of the readily releasable pool (RRP) of glutamate in naïve male and female mice. Cumulative EPSC data following the 100-Hz stimulation train reveal a greater response in females compared to males [main effect of sex, *F*(1,11) = 5.97, *p* = 0.033, *n* = 6–7/group, Fig. 3A]. An analysis of the RRP size revealed that females have a significantly larger readily releasable pool of glutamate compared to males [*t*(10) = 2.39, *p* = 0.038, *n* = 5–7/group, Fig. 3B]. This increase is accompanied by a decrease in release probability, as evidenced by the significantly lower PTrain value seen in females [*t*(10) = 2.61, *p* = 0.026, *n* = 5–7/group, Fig. 3C].

### Biological sex affects LTD induction in the nucleus accumbens core in mice

**Fig. 4:**
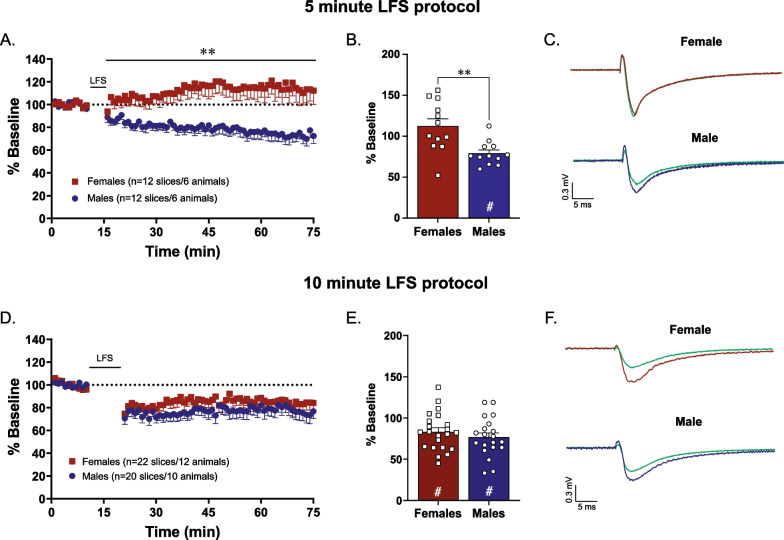
Longer induction protocols are needed to induce LTD in the nucleus accumbens core of female mice compared to male mice. While a shorter low-frequency stimulation protocol (1-Hz train of paired stimuli for 5 min) is sufficient to induce LTD in males, it does not induce LTD in females (**A**; *n* = 12/group). Change in fEPSP slope over 1-h post-LFS shows a significant blunting of LTD in females compared to males in this protocol (**B**; *n* = 12/group). In contrast, a longer low-frequency stimulation protocol (1-Hz train of paired stimuli for 10 min) is sufficient to induce LTD in both sexes (**D**; *n* = 20–22/group). There is no effect of sex on fEPSP slope over 1-h post-LFS in this protocol (**E**; *n* = 20–22/group). Representative pre- and post-LFS traces (**C**, **F**). **p < 0.01 effect of biological sex ^#^*p* < 0.01 significant LTD

We next explored whether LTD in the nucleus accumbens core functions similarly in both sexes. Following a 5 min paired-pulse protocol there is successful LTD induction in male, but not female, mice [*t*(22) = 3.40, *p* = 0.003, *n* = 12/group, Fig. 4A, B]. It is proposed increasing the number of pulses in a LTD protocol can increase the magnitude of depression [61]. Therefore, we doubled the number of pulses to examine whether a longer protocol induces LTD more effectively in female animals. Following a 10-min paired-pulse protocol this difference is abolished as we see a similar magnitude of LTD induction in both sexes [*t*(40) = 0.92, *p* = 0.37, *n* = 20–22/group, Fig. 4D, E].

### PKMζ knockout alters LTD in the nucleus accumbens core in a sex- and protocol-specific manner in mice

**Fig. 5:**
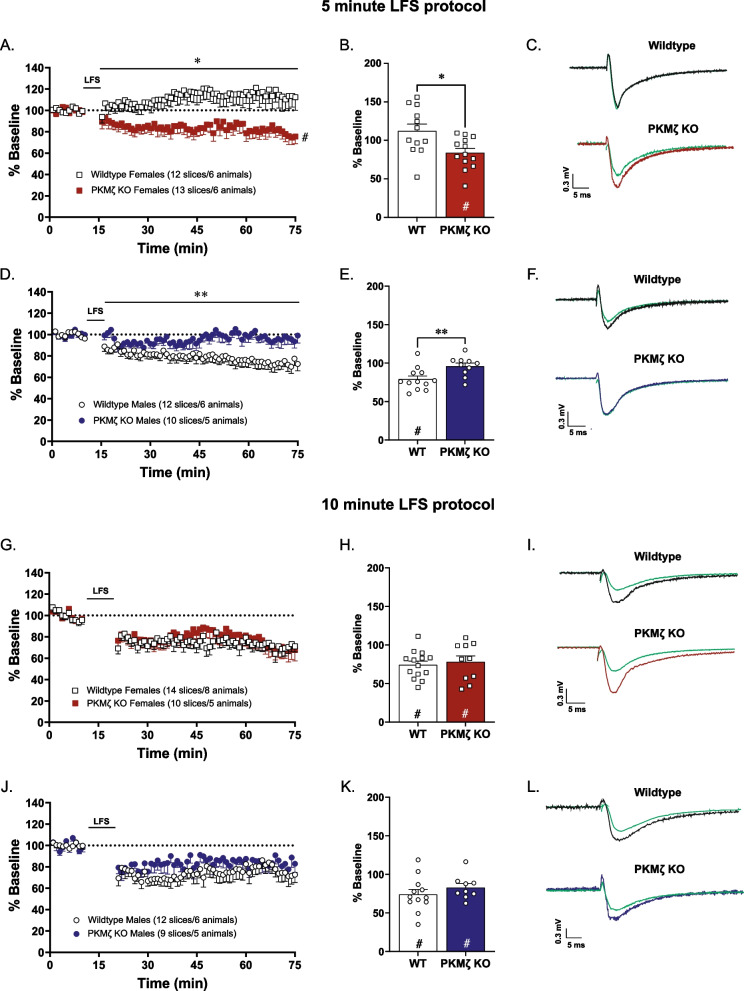
PKMζ knockout alters LTD in mice in the nucleus accumbens core in a sex-specific manner. LTD induction in female mice by a shorter protocol of LFS (1-Hz train of paired stimuli for 5 min) is facilitated in PKMζ knockout animals compared to wildtype controls (**A**; *n* = 12–13/group). Change in fEPSP slope over 1-h post-LFS shows PKMζ knockout facilitates LTD in female mice compared to wildtype controls (**B**; *n* = 12–13/group). LTD induction in male mice by a shorter protocol of LFS (1-Hz train of paired stimuli for 5 min) is blunted in PKMζ knockout animals compared to wildtype controls (**D**; *n* = 10–12/group). Change in fEPSP slope over 1-h post-LFS shows PKMζ knockout blunts LTD in male mice compared to wildtype controls (**E**; *n* = 10–12/group). Following the longer LFS induction protocol (1-Hz train of paired stimuli for 10 min), there is no effect of PKMζ knockout on LTD induction in either male or female mice (**G**; *n* = 10–14/group; **J**; *n* = 9–12/group). Change in fEPSP slope over 1-h post-LFS reveals robust LTD in both wildtype and PKMζ knockout mice (females: **H**; *n* = 10–14/group; males: **K**; *n* = 9–12/group). Representative pre- and post-LFS traces (**C**, **F**, **I**, **L**). *p < 0.05; **p < 0.01 effect of genotype, ^#^*p* < 0.01 significant LTD

Lastly, we aimed to understand whether PKMζ plays a role in LTD within the nucleus accumbens core. While our 5-min paired-pulse protocol does not induce LTD in female wildtype animals, it does in female PKMζ knockout animals [*t*(23) = 2.76, *p* = 0.011, *n* = 12–13/group, Fig. 5A, B]. In males we see the opposite effect where LTD induction is blunted in PKMζ knockout animals [*t*(20) = 2.86, *p* = 0.009, *n* = 10–12/group Fig. 5D, E]. We found the differences between genotypes are abolished in both sexes with a 10-min paired-pulse protocol. There are no significant differences in the magnitude of LTD induction between female wildtype and PKMζ knockout animals [*t*(22) = 0.43, *p* = 0.67, *n* = 10–14/group, Fig. 5G, H] or between male wildtype and PKMζ knockout animals [*t*(19) = 1.02, *p* = 0.32, *n* = 9–12/group, Fig. 5J, K].

## Discussion

Sex differences in the prevalence and presentation of psychiatric diseases are well-established. Despite this fact, the neural mechanisms driving behavioral sex differences in diseases such as SUD and depression are not fully understood. As glutamate transmission influences the development of many psychiatric diseases [1–4], we aimed to understand how glutamatergic transmission within the reward system may differ between males and females. Here, we found baseline sex differences in excitatory transmission at multiple levels within the NAc core. These differences may, in part, underlie some of the established sex differences in psychiatric diseases that involve dysregulated motivation and reward processing.

### Sex differences in postsynaptic glutamatergic transmission

We found in both rats and mice that females have a significantly higher AMPA/NMDA ratio in the NAc core than males. These data indicate heightened synaptic strength in females, an effect that is replicable across multiple species. AMPAR subunits display different kinetic properties with GluA1 homomers having greater conductance than GluA2-containing heteromers [62–69]. As increased excitatory synaptic strength is associated with an increased contribution of GluA2-lacking AMPARs [70], we propose females may have a heightened contribution of GluA1 AMPARs in the NAc compared to males. While we did not investigate receptor distribution here, there are known sex differences in glutamate receptor expression in the PFC [17, 71, 72] and surface expression of GluA1 fluctuates during the estrous cycle in the NAc [73]. An increased AMPAR contribution in females would drive the heightened AMPA/NMDA ratio we see in both species.

It is established the AMPA/NMDA ratio in the NAc is altered following drug use. Chronic cocaine exposure and cocaine and nicotine reinstatement cause an increase in the AMPA/NMDA ratio in the NAc core [74–76]. This effect of chronic cocaine is reversed with *N*-acetylcysteine, a compound that helps restore disrupted glutamate homeostasis [76]. *N*-Acetylcysteine administration also induces lasting reductions in cocaine and heroin reinstatement and seeking [76–80]. The AMPA/NMDA ratio in the NAc is predominantly examined in the context of reward. However, it is known chronic variable stress does not alter the AMPA/NMDA ratio at ventral hippocampal → NAc projections in either sex [81]. Further, α2-adrenoceptor agonism in the NAc shell reduces anxiety but does not alter the AMPA/NMDA ratio [82, 83]. Therefore, heightened synaptic strength as evidenced by the AMPA/NMDA ratio may be of particularly importance for reward-driven behaviors such as drug-taking. As females are more vulnerable than males to many aspects of SUD [5–10, 84], we propose the heightened synaptic strength we found in the NAc of females may, in part, drive female vulnerability to SUD specifically.

While we might expect to see a corresponding sex difference in sEPSC amplitude, we do not. It is possible this is due to the fact that we did not track the estrous cycle in these studies. While we cannot directly compare mEPSCs and sEPSCs, other studies have found effects of sex on mEPSC amplitude in adult animals. This effect is not present in prepubertal animals and is dependent on estrous cycle stage [37, 38, 44]. These findings highlight the likelihood there would be effects of estrous cycle stage on sEPSC amplitude that we are not capturing in these studies. Nonetheless, our results are not the first to find an effect on the AMPA/NMDA ratio without a corresponding effect on sEPSC amplitude. In the basolateral amygdala, pubescent male rats have a higher AMPA/NMDA ratio than pubescent females but there is no significant difference in sEPSC amplitude [85]. In the dorsolateral striatum, high alcohol preference mice exhibit a lower AMPA/NMDA ratio than low alcohol preference mice but there is also no change in sEPSC amplitude [86].

Spontaneous activity is proposed to rely on different mechanisms than evoked activity. As sEPSCs are spontaneous and the AMPA/NMDA ratio is evoked, it is also plausible NAc inputs are less active spontaneously and therefore we do not see an effect of sex on sEPSC amplitude. Despite the lack of effect of sex on sEPSC amplitude, our AMPA/NMDA ratio data indicate females have heightened postsynaptic strength in the NAc. This could, in part, underlie some of the female vulnerability to psychiatric diseases such as SUD. To further investigate sex differences in the reward system, we also examined measures of presynaptic transmission within the NAc.

### Sex differences in presynaptic glutamatergic transmission

We found significant sex differences in presynaptic glutamatergic transmission across two species in the NAc core. In mice, we replicated our previous finding that females have a larger RRP of glutamate than males [45]. Differences in the size of the RRP would lead to a variety of changes in presynaptic transmission, including possible differences in release probability. Therefore, we also examined release probability in mice and found it is lower in females within this region compared to males. We did not see a corresponding effect on sEPSC frequency in either mice or rats. Despite this, our RRP data indicate females may have heightened presynaptic glutamatergic transmission in the NAc.

There are a few explanations for why we see a significant effect of sex on the size of the RRP and release probability but not on sEPSC frequency. First, the size of the RRP in the NAc is likely projection-specific. Our previously published data demonstrate the effect of sex on overall RRP size is not present in projections specifically from the ventral hippocampus [45]. While we found an overall sex difference in the size of the RRP, we are not able to determine whether this effect is driven by specific glutamatergic inputs from the ventral hippocampus. This indicates another input region to the NAc likely drives the large sex difference we found and replicated here. Input-specificity may explain why there is an effect of sex on the RRP but not on sEPSC frequency.

Our sEPSC frequency data are likely affected by the estrous cycle as well, as mEPSC frequency in the NAc core is altered by sex and ovarian hormones [36–38, 42, 44, 87]. sEPSC frequency is also cell-type specific within the NAc. Frequency is significantly higher in D2-containing neurons compared to D1-containing neurons [88]. Differentiating by D1 vs D2 neurons may elicit effects of sex that we did not capture in these studies. Therefore, our effect of sex on the RPP and release probability but not on sEPSC frequency could be due to the estrous cycle, the specific inputs and cell-types involved, or a combination of these. Nonetheless, our RRP and release probability data indicate there are biological sex differences in presynaptic glutamate transmission within the NAc core. These differences may have functional consequences that alter motivation and reward processing.

### Sex differences in LTD

Along with these differences in baseline post- and pre-synaptic glutamate transmission, we also found sex differences in synaptic plasticity within the NAc core. The current study demonstrates that the induction threshold for LTD is higher in females than males, with a shorter paired-pulse protocol inducing LTD in males but not females. LTD was induced in females following a longer paired-pulse protocol. The extent of the LTD in females was similar to that in males at this stimulation duration. LTD is therefore harder to induce in the NAc of females and requires a more intense stimulation protocol than in males.

The sex difference we found in LTD induction is likely a result of the larger RRP in females. LTD induction reduces the size of the RRP and synaptic depression is a result of RRP depletion [89–91]. In females, the larger RRP would make it more difficult to induce synaptic depression. This likely prevents the induction of LTD at shorter protocols and allows induction with longer protocols as they induce enough vesicle release to induce LTD. Therefore, heightened glutamatergic activity in the NAc of females likely makes the region less plastic.

While sex differences in LTD are largely unexplored, it is established LTD is modulated by circulating ovarian hormones [92]. Most work has focused on long-term potentiation (LTP), however. The mechanisms and expression of LTP are modulated by biological sex and sex hormones. LTP in the hippocampus is significantly influenced by fluctuating hormone levels during the estrous cycle [93–96] and male rats exhibit LTP to a broader range of tetani than females [97]. In striatal MSNs from male animals, both aromatase inhibition and ER antagonism can alter LTP induction but neither alter LTD [98]. These data indicate synaptic plasticity can be modulated by biological sex, but the role of sex hormones is likely nuanced. While we did not specifically investigate the role of gonadal hormones here, our data indicate increased excitatory activity in females blunts LTD induction.

### PKMζ has a sex-specific role in LTD

Our findings from both the whole-cell and LTD studies clearly suggest sex differences in the glutamate system. We further investigated these differences by examining the effect of PKMζ knockout on LTD. We found male knockout mice exhibit blunted LTD compared to wildtype controls. The effect of genotype was abolished following a more intense LTD induction protocol. This suggests that PKMζ knockout increased the induction threshold for LTD in male mice rather than eliminating this form of plasticity altogether.

Trafficking of GluA2 AMPAR subunits plays a critical role in LFS-induced LTD [47, 99, 100]. Within the NAc, altering GluA2 trafficking via glutamate receptor-interacting protein (GRIP) knockout abolishes LTD [101]. GRIP works to stabilize GluA2-containing AMPARs to the synapse, similarly to PKMζ. PKMζ potentiates NSF-mediated insertion of GluA2-containing AMPARs into the cell membrane [52] and cytosolic levels are decreased in the hippocampus following LTD induction [102]. The results found here demonstrate a definitive role for PKMζ in LTD within the NAc. Our data further the evidence that AMPAR trafficking proteins play a crucial role in LTD.

In contrast to the effect of PKMζ knockout in males, we found that PKMζ knockout had the opposite effect in female mice, decreasing the threshold for LTD induction. While the shorter induction protocol did not elicit LTD in wildtype females, it did successfully induce LTD in PKMζ knockout females. As previously mentioned, the larger RRP we found in wildtype females explains the sex difference we see in LTD. Though the role of PKMζ is thought to be exclusively postsynaptic, it is possible PKMζ is altering presynaptic activity in females in a manner that blunts synaptic plasticity. As most studies examining PKMζ activity used male animals, this effect may not be the same across both sexes.

This is the first study to examine LTD in constitutive PKMζ knockout mice in both sexes. However, previous studies have explored the effect of constitutive PKMζ knockout on LTP. Within the hippocampus, PKMζ knockout animals exhibit normal LTP and there are no effects of sex [60]. While this suggests that PKMζ is not necessary for hippocampal LTP, it is not known whether this reflects regional differences in the role of PKMζ or differences in the mechanisms driving these different forms of plasticity. Our results indicate PKMζ alters LTD and does so in a sex-specific manner. Future studies could further characterize the role of PKMζ in glutamatergic transmission by examining measures such as AMPA/NMDA ratio and sEPSCs in knockout mice.

Our study is not the first to find sex-specificity in PKMζ activity. Behaviorally, constitutive PKMζ knockout reduces anxiety-like behavior in males but not females [58] and site-specific deletion of PKMζ in the NAc potentiates cocaine-taking exclusively in male mice [53]. Following exposure to cocaine and methamphetamine there are also sex-specific alterations to PKMζ expression [53, 103]. Our data indicate PKMζ also plays a sex-specific role in LTD within the NAc. In addition to sex-specificity, we also found the effect of PKMζ knockout on LTD to be “dose” specific as the effect of genotype is not apparent following the stronger LTD induction protocol. This further highlights the need to examine multiple induction protocols as sex differences in plasticity may be quantitative rather than qualitative.

## Perspectives and significance

Here, we show there are baseline sex differences in excitatory transmission within the NAc core. In combination with our previously published data in the mPFC, we propose these sex differences may, in part, underlie the behavioral sex differences seen in psychiatric diseases such as SUD and depression. These data highlight the importance of considering sex as a biological variable in the development of pharmacotherapies for psychiatric disease. Targeting different mechanisms in males and females based on sex differences such as the ones shown here could help increase treatment efficacy long-term.

## Conclusions

Altogether, our data demonstrate there are sex differences in synaptic plasticity at multiple levels within the reward system. Altered glutamate transmission is involved in multiple psychiatric diseases, therefore we propose baseline sex differences in synaptic plasticity within this region may drive some of the behavioral sex differences in these illnesses. Though we did not track estrous cycle in female animals here, it is likely many of the effects we see are mediated by natural hormonal fluctuations. Nonetheless, these data highlight the need for further exploration of baseline sex differences in the mechanisms driving psychiatric disease. Greater understanding of these mechanisms will allow for the development of more targeted, and ideally more effective, pharmacotherapies to treat diseases such as SUD and depression.

## Data Availability

The datasets used and/or analyzed during the current study are available from the corresponding author on request.
